# Understanding the Interactions Between Driving Behavior and Well-being in Daily Driving: Causal Analysis of a Field Study

**DOI:** 10.2196/36314

**Published:** 2022-08-30

**Authors:** Paul Stephan, Felix Wortmann, Kevin Koch

**Affiliations:** 1 Bosch IoT Lab Institute of Technology Management University of St Gallen St Gallen Switzerland

**Keywords:** well-being, daily driving, causal inference, commute, field study, directed acyclic graph, just-in-time interventions, mental well-being, stress, mental health

## Abstract

**Background:**

Investigating ways to improve well-being in everyday situations as a means of fostering mental health has gained substantial interest in recent years. For many people, the daily commute by car is a particularly straining situation of the day, and thus researchers have already designed various in-vehicle well-being interventions for a better commuting experience. Current research has validated such interventions but is limited to isolating effects in controlled experiments that are generally not representative of real-world driving conditions.

**Objective:**

The aim of the study is to identify cause–effect relationships between driving behavior and well-being in a real-world setting. This knowledge should contribute to a better understanding of when to trigger interventions.

**Methods:**

We conducted a field study in which we provided a demographically diverse sample of 10 commuters with a car for daily driving over a period of 4 months. Before and after each trip, the drivers had to fill out a questionnaire about their state of well-being, which was operationalized as arousal and valence. We equipped the cars with sensors that recorded driving behavior, such as sudden braking. We also captured trip-dependent factors, such as the length of the drive, and predetermined factors, such as the weather. We conducted a causal analysis based on a causal directed acyclic graph (DAG) to examine cause–effect relationships from the observational data and to isolate the causal chains between the examined variables. We did so by applying the backdoor criterion to the data-based graphical model. The hereby compiled adjustment set was used in a multiple regression to estimate the causal effects between the variables.

**Results:**

The causal analysis showed that a higher level of arousal before driving influences driving behavior. Higher arousal reduced the frequency of sudden events (*P*=.04) as well as the average speed (*P*=.001), while fostering active steering (*P*<.001). In turn, more frequent braking (*P*<.001) increased arousal after the drive, while a longer trip (*P*<.001) with a higher average speed (*P*<.001) reduced arousal. The prevalence of sunshine (*P*<.001) increased arousal and of occupants (*P*<.001) increased valence (*P*<.001) before and after driving.

**Conclusions:**

The examination of cause–effect relationships unveiled significant interactions between well-being and driving. A low level of predriving arousal impairs driving behavior, which manifests itself in more frequent sudden events and less anticipatory driving. Driving has a stronger effect on arousal than on valence. In particular, monotonous driving situations at high speeds with low cognitive demand increase the risk of the driver becoming tired (low arousal), thus impairing driving behavior. By combining the identified causal chains, states of vulnerability can be inferred that may form the basis for timely delivered interventions to improve well-being while driving.

## Introduction

With rising numbers of mental disorders worldwide, maintaining well-being has become an important public health issue [1], with a special focus on untreated cases [2]. In recent years, there has been an increased interest in investigating ways to improve well-being in everyday situations in order to prevent mental disorders [3,4]. Especially interventions aimed at improving well-being in moments when a person is susceptible to a deteriorating mental state, a so-called *state of vulnerability*, have shown great promise [5,6]. In this regard, the just-in-time adaptive intervention (JITAI) framework has recently gained major attraction as it guides researchers in their way to develop effective interventions that are delivered at the right time and in the right situation. To implement such a JITAI effectively, a profound understanding of the contextual factors leading to a state of vulnerability is necessary [7].

A suitable everyday situation in which an intervention may promise beneficial effects on well-being is daily driving [8]. Daily car commuters spend a considerable amount of time on the street every day, often associated with events causing frustrations and loss of time, such as caused by traffic jams, congestion, and unpredictability [9,10]. Kahneman et al [11] found the daily commute to be one of the least pleasant activities of the day. Accordingly, states of deteriorating well-being are likely to occur in daily driving. Simultaneously, the car is a suitable place for JITAIs as there are multiple sensors to detect the current driving conditions (eg, lane or traffic object detection [12,13]) and driver states (eg, arousal states [14] or emotions [15,16]) as well as to deliver interventions using advanced multimedia systems.

Recent work investigated when drivers are interruptible by [17,18] or even responsive to interventions [19] while driving. Moreover, researchers designed and validated the effect of well-being interventions that can be conducted while driving, for example, breathing exercises [20] and music or mindfulness experiences [21]. According to the JITAI framework, interventions are most effective when triggered in a state of reduced well-being [7]. To identify such states of vulnerability and thereby improve road safety [22], the factors influencing well-being while driving must be determined. Since well-being likely influences driving behavior and vice versa, a thorough understanding of the causal relationships is crucial for evaluating the driver’s mental state. Therefore, this study examines the interactions between driving behavior and well-being during daily driving.

Because drivers are exposed to a variety of contextual factors, it is difficult to establish robust causal relationships based on existing statistical analysis [23]. Previous studies on driving and well-being have been primarily limited to isolating specific relationships in simulation experiments [24-28]. However, this controlled environment limits the results as stimuli are artificially induced, and thus effects do not generalize well to the wide range of situations encountered in everyday road traffic [29-31]. To thoroughly understand the relationship between driving and well-being, it seems necessary to study real-world data using novel methods for causal analysis.

The aim of this analysis is to unveil a robust causal architecture, that is, the underlying network of causes and effects [32], between driving behavior and well-being. We applied novel causal inference algorithms to derive these relationships from complex observational data collected in a real-world driving study, in which we investigated drivers’ well-being over a period of 4 months. The derived causal architecture forms a basis for inferring states of vulnerability that can be targeted by digital interventions.

## Methods

### Field Study Setting and Variables

The data were gathered in a field study in which we handed over to 10 participants between the ages of 26 and 55 years a car each for daily driving. For maximizing external validity, we selected a broad spectrum of typical daily commuters with different demographics, life and family situations, and driving habits (purposive sampling). Detailed information about the participants is documented in Multimedia Appendix 1. Participants completed most of their driving in their residence area, which for all of them was the region around Stuttgart (Germany). The field study lasted for a period of 4 months, from July to November 2019.

To measure a wide variety of factors that could impact the well-being of the driver, we retrofitted the study cars for data collection. The participants self-assessed their current emotional state before and after driving based on questionnaires by a smartphone mounted next to the multimedia system. Moreover, we installed in every car a data collection system that recorded various variables from the vehicle in high frequency (eg, the steering wheel speed, brake pedal and gas pedal positions, or the Global Positioning System [GPS] location) to measure the driving behavior as well as the vehicle state. Our final data set comprised 13 variables that were classified into 4 categories: emotions, driving behavior, trip-dependent factors, and predetermined factors. We chose these 4 categories based on related work examining driving behavior and well-being [19,21]. A detailed list of the included variables can be found in Multimedia Appendix 2. This set of variables provides a comprehensive exploratory basis for understanding how driving behavior and well-being relate to each other. We explain these categories in the following paragraphs.

The *emotions* of the driver were assessed according to the circumplex model of affect, which is composed of the 2 dimensions *arousal* and *valence* [33,34]. The arousal dimension describes the drivers' feeling of being awake, and the valence dimension indicates the corresponding level of happiness. Before and after each drive, the driver indicated their state of arousal and valence on a scale from 0 (very low) to 100 (very high) using the *Affective Slider* [35], which is depicted in Multimedia Appendix 3.

We measured the *driving behavior* using the sensors that each car was equipped with. To quantify the driving behavior, we analyzed the steering and braking behavior of the driver. The *steering* *behavior* was quantified as the proportion of the trip in which the driver was turning the steering wheel. Analogously, the *braking* *behavior* reflected the ratio of the seconds in which the brake pedal was engaged to the total duration of the trip. To account for the risk that a driver takes, we included the frequency of sudden accelerations, sudden braking, or sudden steering as the variable *sudden events*. An event was classified as sudden when the acceleration or steering angle exceeded a prespecified threshold. To identify these events, we used the same approach as in prior work [19] based on the peak detection algorithm from the Python package SciPy [36].

Since the field study was conducted in an uncontrolled setting, we needed to account for contextual and environmental factors that drivers experience. We distinguished between *trip-dependent factors*, which are related to the drive itself, and *predetermined factors*, which are explicitly known to drivers before starting. The *trip-dependent factors* comprise information about the *length* of the trip in seconds and the average *speed* in kilometers per hour. In addition, the *flow* of the trip quantifies the ratio of the actual speed to the potential maximum speed throughout the trip. By combining these 3 factors, we aimed at representing the built environment in which the trip takes place [37]. For example, urban driving will likely result in short trips at low speeds with low flow.

The included *predetermined* *factors* in the causal model were the weather, quantified by the minutes of *sunlight* in the hour that the drive started and whether the trip was or was not performed on the *weekend*. Furthermore, we recorded whether another *occupant* was present and whether the trip was a *commute* between home and work, as derived from the GPS location at the beginning and end of each trip. To reduce skewness and to establish a common scale across all continuous variables, we standardized the data to a mean of 0 and an SD of 1.

### Ethical Considerations

This field study was reviewed by the Institutional Review Board of the University of Bern, Switzerland (approval #2019.04-00003).

### Establishing a Causal Relationship

**Figure 1:**
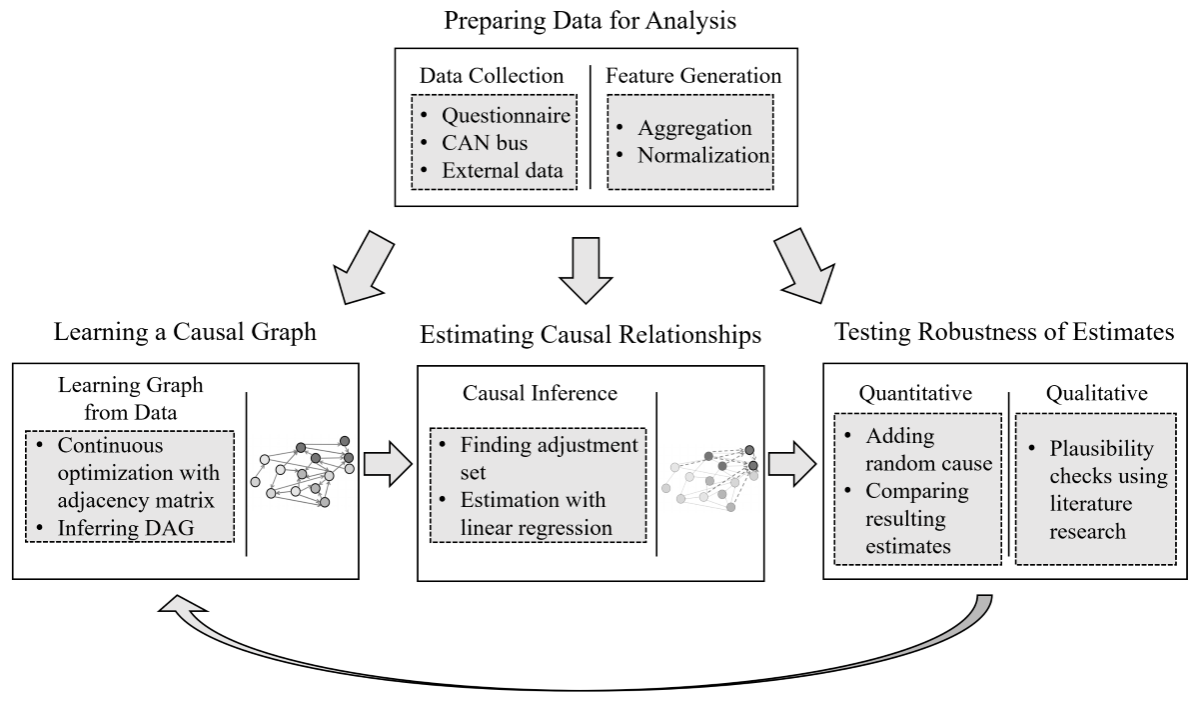
Our workflow for causal analysis. CAN: controller area network (car sensor data); DAG: directed acyclic graph.

We conducted a field study to examine well-being in real-world driving situations, while maximizing the generalizability of our results. However, this purely observational study design comes at the cost of controllability. Therefore, the interactions between factors of well-being and driving behavior cannot be directly estimated as in a controlled experiment. Instead, we propose a framework for causal inference based on a causal directed acyclic graph (DAG), which visually represents the causal architecture formed by all recorded variables [38-68]. Our workflow for causal inference was designed as a 3-step process, as illustrated in Figure 1. Detailed theoretical information about the workflow and the causal methodology can be found in Multimedia Appendix 4.

The causal DAG was constructed using the *DAG with NOTEARS* algorithm [69] on the data of the field study. This algorithm performs continuous optimization on a matrix representation of the graph rather than using constraint-based or local methods for inferring a graphical model. Thereby, a single graph maximizing the score function of the algorithm is found. We used the resulting graph to determine paths, which depict relationships between variables. To isolate the effect of one variable on another, the paths carrying spurious associations must be eliminated, while preserving the paths that transmit the causal effect. We isolated the relevant paths by applying the *backdoor criterion* [47], which identifies the set of variables that need to be controlled. This so-called *adjustment set* was subsequently used in a multiple regression to identify the causal effect between the variables of interest.

For determining the robustness of the resulting estimates, the effect was recalculated in a DAG with an added random confounder [65]. More specifically, a difference between the original and the new estimate close to 0 indicates that an effect is robust to unobserved confounders. Moreover, this test indicates the robustness of the estimate against a potential violation of the linear regression assumptions. Additionally, trivially impossible effects, such as an effect from arousal after to before driving, were a priori excluded from the causal DAG. A full list of excluded effects can be found in Multimedia Appendix 5.

## Results

### Descriptive Results

The 10 participants completed on average 163.8 trips (SD 89.28) during the 4 months of the study. The mean duration of a trip was 29 minutes (SD 20), and an average participant drove 19.5 km (SD 28.15). Our data set appears to cover typical daily driving, as the drivers followed a large variety of routes both in urban and in rural areas. Of the 1638 trips, 1343 (82%) took place during the week. In addition, 393 (24%) of the trips were labeled as a commute, and 1245 (76%) were drives to frequently visited destinations. On all these trips, the participants completed the affective slider. The affective slider results before driving were on average 73.66 (SD 18.24) for arousal and 69.76 (SD 16.74) for valence and after driving were on average 71.53 (SD 19.58) for arousal and 69.26 (SD 16.91) for valence.

### Causal Analysis

To understand which factors impact well-being, we conducted a causal analysis based on the causal DAG learned from data, which can be found in Multimedia Appendix 6. The causal effect sizes, hereinafter abbreviated as *CE*, describe the impact of a 1-SD change in the source variable on the target variable. As all variables were standardized and to facilitate comparisons, the resulting effect size is also given in SDs. The statistically significant (α=.05) causal effects grouped by origin and target nodes are listed in Table 1. The robustness test reports the difference between the causal estimate from the analysis and the causal estimate when adding a random confounder to the model. A value close to 0 indicates robust causal estimates.

**Table 1:** Results of our causal analysis.

Source			Target		CE^a^		95% CI		*P* value		Robustness test
**Emotions on driving behavior**											
	Before arousal	Steering		0.13		0.09- 0.16		<.001		−0.00003	
	Before arousal	Sudden events		−0.06		–0.11 to 0.02		.04		0.00031	
	Before arousal	Speed		−0.11		–0.13 to –0.07		.001		−0.00104	
**Driving behavior on emotions**											
	Braking	After arousal		0.10		0.06-0.14		<.001		−0.00089	
	Speed	After arousal		−0.17		–0.20 to –0.12		<.001		0.00001	
**Predetermined factors on emotions**											
	Sun	Before arousal		0.12		0.08-0.18		<.001		−0.00048	
	Sun	After arousal		0.14		0.11-0.18		<.001		0.00016	
	Occupants	Before valence		0.38		0.26-0.49		<.001		−0.00008	
	Occupants	After valence		0.37		0.24-0.51		<.001		0.00021	
**Trip-dependent factors on emotions**											
	Length	After arousal		−0.13		–0.45 to –0.06		.001		−0.00073	
**Emotions on emotions**											
	Before arousal	Before valence		0.18		0.14-0.22		.002		−0.00084	
	Before arousal	After arousal		0.74		0.70-0.76		<.001		0.00000	
	Before arousal	After valence		0.19		0.15-0.24		.01		0.00009	
	Before valence	After valence		0.77		0.74-0.80		<.001		−0.00161	
	After valence	After arousal		0.13		0.08-0.18		<.001		0.00013	

^a^CE: causal effect size.

For developing a better understanding of the interaction between driving and well-being, we investigated the effects in both directions (ie, well-being on driving and driving on well-being). In the following paragraphs, we report on the significant results (α=.05). All effects that we discuss are highly robust with respect to omitted variables and to violations of the linear regression assumptions as the change in the causal estimate is smaller than 0.001 after adding a random confounder.

### Effects Related to Well-being Before Driving

**Figure 2:**
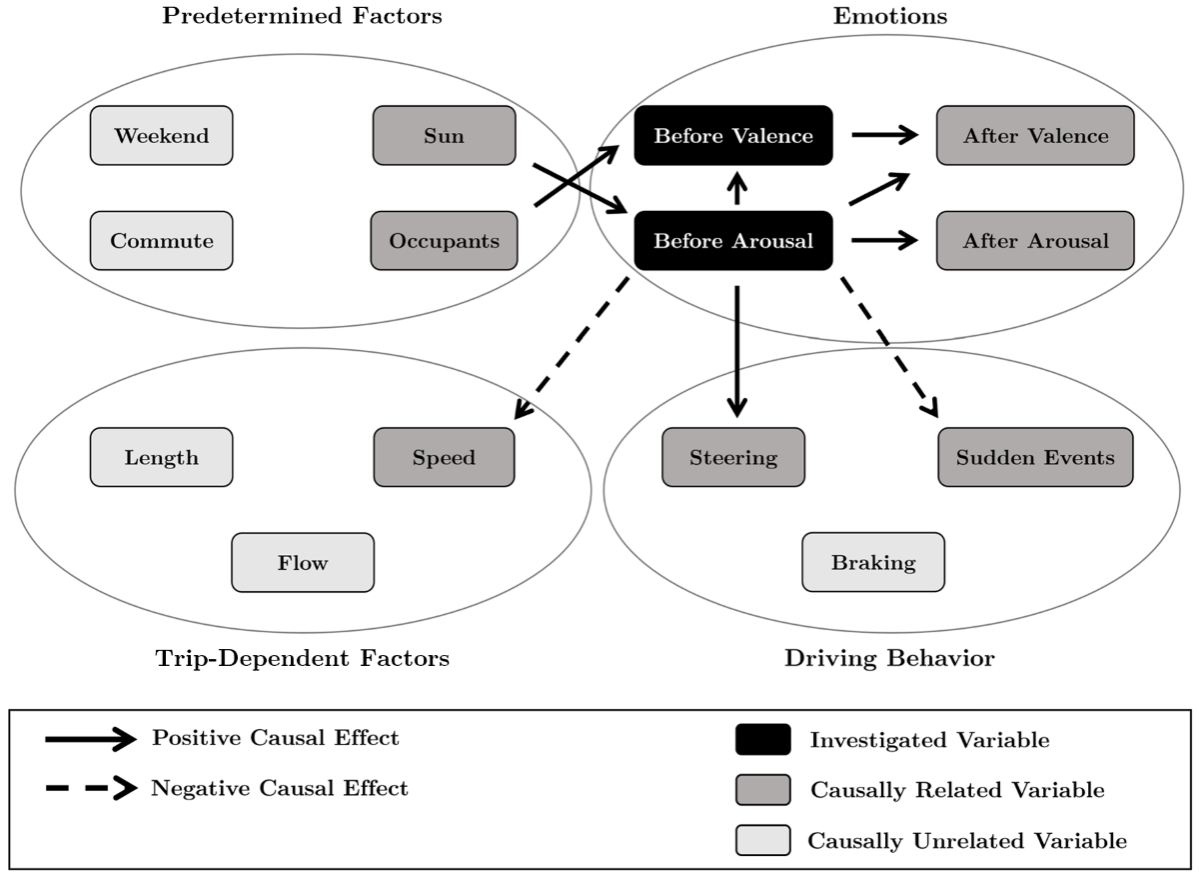
Causal effects regarding well-being before driving.

Figure 2 shows all causal effects related to well-being before driving. The analysis showed that before-driving emotions cause changes in the driving behavior as well as in trip-dependent factors. Regarding behavioral variables, a higher level of *before-driving arousal* significantly increased the frequency of *steering* (CE=0.13, *P*<.001) and decreased the occurrence of *sudden events* (CE=−0.06, *P*=.04). More specifically, these effects mean that an increase of 1 SD of arousal prevented 8 sudden events per hour. Moreover, higher *before-driving arousal* decreased the *speed* of trips (CE=−0.11, *P*=.001), which amounted to 2 km/hour per 1 SD of arousal. Moreover, a significant interaction between emotions existed. *Before-driving arousal* positively influenced the level of *before-driving valence* (CE=0.18, *P*=.002). *Before-driving valence* had no statistically significant effects on driving behavior or on trip-dependent factors.

Before-driving emotions were also influenced by predetermined factors. The presence of *occupants* caused an increase in *before-driving valence* (CE=0.38, *P*<.001), and more *sunlight* caused higher levels of *before-driving arousal* (CE=0.12, *P*<.001). The variable *weekend* had no causal impact on the *before-driving valence* or the *arousal* of the participants.

### Effects Related to Well-being After Driving

**Figure 3:**
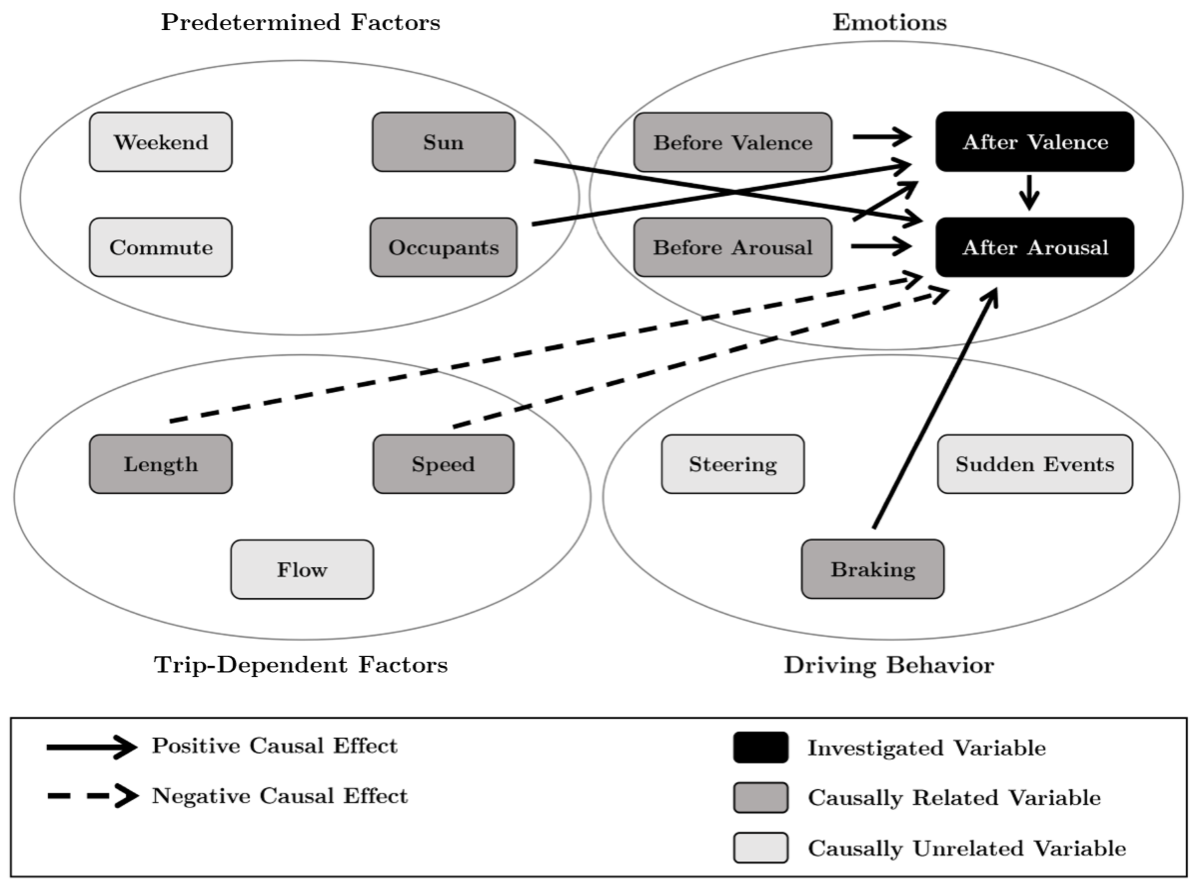
Causal effects regarding well-being after driving.

Figure 3 shows the causal effects related to well-being after driving. The emotions after driving a car are influenced by the driving behavior as well as by trip-dependent and predetermined factors. Variables related to actual driving showed that both higher average *speeds* (CE=−0.17, *P*<.001) as well as longer *trips* (CE=−0.13, *P*=.001) caused lower levels of *arousal*. Moreover, driving behavior had an influence, with more frequent *braking* increasing the *after-driving arousal* (CE=0.1, *P*<.001).

Analogously to the effects of predetermined factors before the trip, *sunlight* increased *after-driving arousal* (CE=0.14, *P*<.001) and the presence of *occupants* increased *after-driving valence* (CE=0.37, *P*<.001). Thus, the effect of *sunlight* on *arousal* was stronger after than before driving, whereas the effect from *occupants* on *valence* was smaller after driving.

The emotions before starting the trip strongly influenced the emotions after having completed the trip. This relationship was especially evident when examining the causal effects from *before-driving* to *after-driving arousal* (CE=0.74, *P*<.001) and *valence* (CE=0.77, *P*<.001). Further, a significant interaction existed between *emotions*, with the *before-driving arousal* influencing the level of *after-driving valence* (CE=0.19, *P*=.005). In addition, higher *after-driving arousal* states causally increased *after-driving valence* (CE=0.13, *P*<.001).

## Discussion

### Principal Findings

The results of our field study indicate that well-being significantly influences driving behavior and vice versa. Moreover, we found effects from predetermined and trip-dependent factors on valence. In the following paragraphs, we highlight our findings and contextualize them with potential explanations.

We found a significant impact of arousal on several driving behavior variables. With higher levels of arousal, drivers had fewer sudden maneuvers, steered more, and drove at lower speeds. We explain these effects with improved alertness due to high arousal [70]. More alert drivers react faster and in a more controlled manner to unexpected events. Therefore, they can proactively avoid sudden driving maneuvers, which reduces the risk of accidents [24]. Moreover, this anticipatory driving behavior with higher arousal leads to more steering and is potentially a sign of active control of the vehicle. Due to alertness and anticipatory driving, drivers may also proactively adapt the speed of the vehicle earlier to changing driving situations, which results in lower speeds.

For the inverse relationship, we found significant effects showing that driving-related factors impact the arousal of drivers. First, the higher the average speed was, the lower the after-driving arousal state was. Second, we found that the length of the trip negatively influences arousal. Since this deterioration of arousal is counteracted by frequent braking, we assume that monotonous driving situations (ie, long trips at high speeds with no need to brake frequently) cause a decrease in arousal. Cognitive tasks, such as braking, seemed to interrupt the perceived monotony and, thus, reduced the negative effect on arousal throughout the trip.

In contrast, we could not identify any statistically significant effect between valence and driving. The missing impact of low flow or sudden events on valence may be explained by the high driving experience of the participants, who may have grown accustomed to these conditions (eg, daily experience of traffic jams on commutes). However, the lack of effects may also be explained by a possible transient impact of adverse events, such as a traffic jam or consecutive red lights. After having reached the destination, these occurrences may have been forgotten and other thoughts may determine the disclosed end-of-trip valence. Further studies should evaluate the immediate impact of adverse conditions on valence.

Besides the actual driving, we identified predetermined factors that influenced well-being. First, more sunlight (ie, better weather) increased before- and after-driving arousal. Sunlight is known to impact daily mood in general and to reduce tiredness [71]. Second, the presence of occupants increased before- and after-driving valence. The explanation of this effect may be that social interaction is associated with a better sense of well-being [72]. In contrast, occupants had no influence on the arousal of drivers. Although occupants may reduce the monotony of a drive, the social interactions may also lead to social fatigue [73] and thus limit a potential arousal improvement.

Furthermore, we found significant interactions between the dimensions of well-being. The levels of arousal and valence before driving were highly correlated with the respective levels after driving. Most likely, carry-over effects occur, for example, awake drivers are still more awake at the end of the trip than drivers who already started while feeling tired. Moreover, both well-being dimensions are positively associated with each other. Building upon our prior reasoning, we propose 2 potential explanations. First, alert drivers experience fewer adverse events and therefore feel more positive by the end of the trip. Second, higher valence can make drivers more resistant to boredom, which reduces the feeling of monotony.

### Comparison With Prior Work

This study aimed at investigating the complex causal architecture of well-being in daily driving using a real-world, uncontrolled field study. In contrast, previous studies have mainly focused on isolating specific effects in controlled experiments (eg, simulator studies). In the following paragraphs, we compare the significant effects between daily driving and well-being of our exploratory study to prior driving studies. These studies serve hereby as a plausibility check of our findings.

Our explanation of the positive impact of increased arousal on driving behavior is in line with the prior literature. Corfitsen [74] found in a survey combined with a reaction time test that low arousal states (ie, fatigue) are a major cause of longer reaction times while driving at night. Moreover, McGehee et al [24] showed in an experimental study on a test track that these longer reaction times are a major risk factor for accidents. However, our findings concerning valence differ from the previous literature. Prior simulator studies have revealed a significant negative effect of extreme valence states (very happy and very unhappy) on driving behavior [25]. The lack of effects of valence in our study could be explained by the setting of the field study. Whereas in the simulator study [25], strong valence-changing stimuli were induced, our study aimed to collect data on everyday driving situations with less strong valence changes.

Furthermore, we find confirmation that monotonous driving reduces the arousal of drivers. Thiffault and Bergeron [26] observed in a simulator study that continuous driving without any external stimulus induces fatigue and tiredness, which increases with time. Moreover, our conclusion that arousal levels are reduced by driving at high speeds due to the monotonous setting is supported by a simulator study by Ting et al [27]. Their study showed that highway driving leads to fatigue, which negatively affects driving performance and increases the risk of accidents, as priorly discussed [24,71]. We can further confirm that cognitive tasks, such as frequent braking, improve after-driving arousal. The results of a simulator experiment by Dunn and Williamson [28] showed that cognitive demand mitigates monotony.

### Implications for Intervention Research and Practice

Our findings can be used to allow for more effective JITAIs by providing an estimate for when drivers are likely at risk of feeling tired or unhappy, that is, when they are in a state of vulnerability. By improving the well-being of the driver, such interventions have the potential to increase road safety and reduce the frequency of accidents.

Interventions for increasing well-being while driving can be conceptualized in 2 ways. First, the causes for states of vulnerability can be directly targeted according to our causal architecture. For instance, the findings indicate that a long drive with little braking and steering generates a monotonous driving situation, which sets the driver at risk of a state of low arousal. Second, interventions can react to detected states of vulnerability. For instance, if an increased number of sudden events, less steering, or increased speeds are recognized, it is an indication that the arousal of the driver has decreased. Thus, an intervention could be triggered that acts as a mental stimulus to increase arousal and thereby prevent drowsy driving. Past research developed and evaluated such interventions, for example, using highly personalized music playlists [21] or gamified driving challenges [75]. Our findings could pave the way for the ideation of new interventions. For instance, valence can be raised with an intervention that leads to social interaction, for example, by recommending calling someone during a break. As another example, a driver’s arousal deterioration due to high-speed driving could be addressed by reminding drivers about the speed limit.

### Strengths and Limitations

We identified relationships between well-being and driving from a 4-month longitudinal field study on real roads with a sample representative of a wide range of commuters. To derive robust relationships, we applied causal inference methods in our analysis. This overall approach has multiple benefits. First, we observed in our study setup the true emotions participants experienced while they were driving. Contrary to laboratory experiments, which often induce or inspect single isolated effects, our results reflect realistic driving situations and therefore generalize better to the real world. Second, our findings can serve as a basis for delivering interventions to react to well-being changes impacting driving behavior. Finally, all identified effects are explainable by the prior literature and robust to violations of assumptions. Therefore, our study serves as a practical example that inferring causal architectures from observational field studies is feasible and may provide insights that go beyond the capabilities of controlled experiments.

The exploratory design of the study comes with some limitations. The analysis was conducted on the aggregated data set of trips during a longitudinal field study of naturalistic driving on public roads. Hence, it does not regard variation between drivers on a personal level. However, by combining experiences from 10 drivers, we could examine interactions that are present across multiple individuals. Further research could include a psychological analysis on the personal level and could combine valence and arousal to construct more complex emotions. Regarding the causal methodology, the *DAG with NOTEARS* algorithm does not definitely guarantee a precise causal DAG and is especially sensitive with respect to the scale of the variables [63]. We mitigated this issue by standardizing all continuous variables and by introducing a random confounder for testing the robustness of the estimates. Further research should establish a framework for assessing the robustness of the causal DAG itself.

### Conclusion

In this paper, we unveiled the complex causal architecture of well-being in daily driving in a real-world field study. Daily driving is a complex setting in which many contextual and personal factors interact. In a real-world field study, this complexity can be replicated more adequately than in a controlled experiment. However, in observational studies, an elaborate causal methodology is necessary for identifying causal effects. Our study identified that arousal is more susceptible to changes while driving than valence. Especially monotonous driving situations, such as long drives on a highway without the need to decelerate or steer frequently, set the driver at risk of becoming more tired. This tiredness impairs driving behavior and can be seen as a state of vulnerability that can be utilized as a trigger for interventions. The knowledge about robust causal effects between well-being and driving behavior can therefore be applied as a basis for deciding when to initiate an intervention to improve the well-being of the driver.

## Appendix

Multimedia Appendix 1 Description of participants.

Multimedia Appendix 2 Further information about variables.

Multimedia Appendix 3 Well-being questionnaire.

Multimedia Appendix 4 Detailed methodology.

Multimedia Appendix 5 A priori excluded direct effects.

Multimedia Appendix 6 Data-based causal directed acyclic graph.

## Abbreviations

CE: causal effect size
DAG: directed acyclic graph
GPS: Global Positioning System
JITAI: just-in-time adaptive intervention

## Data Availability

The raw data of the field study are available upon request, which should be sent to the corresponding author (PS). Any requests will be reviewed by the scientific study board leading the involved research group at the University of St. Gallen. Applications should outline the intended purpose of the data transfer. Only applications for noncommercial use will be considered. All data shared will be anonymized.
