# Supplementary material for: Understanding the Interactions Between Driving Behavior and Well-being in Daily Driving: Causal Analysis of a Field Study
Source: J Med Internet Res. 2022 Aug 30;24(8):e36314. doi: 10.2196/36314 (PMC9472037; doi:10.2196/36314)
Supplement: Multimedia Appendix 1 [file jmir_v24i8e36314_app1.doc]

# Multimedia Appendix 1: Description of Participants

The table below shows descriptive statistics of the driving behavior and routines of the study participants that they reported prior to the study.

| **n** | **Age (years)** | **Gender** | **Residence** | **Driving per week (km)** | **Driving days per week** | **Distance to work (km)** | **Driving experience (years)** |
| --- | --- | --- | --- | --- | --- | --- | --- |
| 10 | M = 37.2  SD = 8.4  min = 26 max = 55 | Female = 4  Male = 6 | Urban = 3  Rural = 7 | M = 327.0  SD = 103.4  min = 100  max = 500 | M = 6.8  SD = 0.4  min = 6  max = 7 | M = 22.6  SD = 7.0  min = 14  max = 35 | M = 20.2  SD = 8.1  min = 9  max = 38 |
