# Supplementary material for: Understanding the Interactions Between Driving Behavior and Well-being in Daily Driving: Causal Analysis of a Field Study
Source: J Med Internet Res. 2022 Aug 30;24(8):e36314. doi: 10.2196/36314 (PMC9472037; doi:10.2196/36314)
Supplement: Multimedia Appendix 2 [file jmir_v24i8e36314_app2.doc]

## Multimedia Appendix 2: Further Information on Variables

## The table below provides further description of each variable as well as the way we quantified each variable from our field study data.

| **Category** | **Variable Name** | **Explanation** | **Measurement** |
| --- | --- | --- | --- |
| Emotions | Before Arousal | Perceived level of arousal before trip | Scale from 0 (very low) to  100 (very high) |
|  | Before Valence | Perceived level of valence before trip | —“— |
|  | After Arousal | Perceived level of arousal after trip | —“— |
|  | After Valence | Perceived level of valence after trip | —“— |
| Driving Behavior | Steering | Frequency of steering | Ratio of time spent steering  to total time |
|  | Braking | Frequency of braking | Ratio of time spent braking  to total time |
|  | Sudden Events | Frequency of sudden events | Aggregate of sudden  acceleration, deceleration,  and steering per minute |
| Trip Characteristics | Length | Duration of trip | Seconds |
|  | Speed | Average speed of trip | Kilometers per hour |
|  | Occupants | Presence of passengers | Dichotomous variable as  measured by sensors |
|  | Commute | Trip is the way between workplace and home and vice versa | Dichotomous variable  identified using GPS data |
|  | Flow | Measure of continuity of driving | Ratio of actual speed to  speed limit |
| External Conditions | Sun | Duration of sunshine | Duration of sunshine  in starting hour |
|  | Weekend | Day of trip is on weekend | Dichotomous variable  using trip date |
