# Supplementary material for: Understanding the Interactions Between Driving Behavior and Well-being in Daily Driving: Causal Analysis of a Field Study
Source: J Med Internet Res. 2022 Aug 30;24(8):e36314. doi: 10.2196/36314 (PMC9472037; doi:10.2196/36314)

# Multimedia Appendix 3: Well-being Questionnaire

now

The figure below shows our Affective Slider implementation participant responded throughout the study before and after driving on a study smartphone that was retrofitted to the car.

now


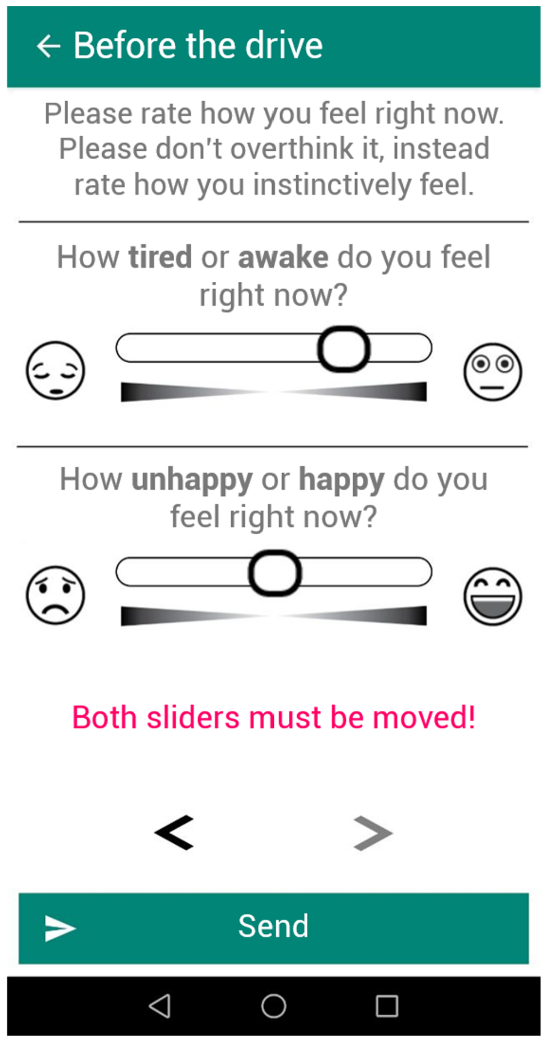

Supplement: Multimedia Appendix 3 [file jmir_v24i8e36314_app3.doc]
