# Supplementary material for: Understanding the Interactions Between Driving Behavior and Well-being in Daily Driving: Causal Analysis of a Field Study
Source: J Med Internet Res. 2022 Aug 30;24(8):e36314. doi: 10.2196/36314 (PMC9472037; doi:10.2196/36314)
Supplement: Multimedia Appendix 4 [file jmir_v24i8e36314_app4.doc]

# Multimedia Appendix 4: Detailed Methodology

This supplementary document provides detailed information on the theoretical background and practical implementation of the causal methodology applied for establishing cause-effect relationships from our field study.

## Causal Graphs for Causal Inference

Causal graphs visually represent cause-effect relationships by depicting variables as nodes and causal dependencies as edges joining the nodes [38]. Each edge shows a direct causal effect between the respective source and target variable. Effects are propagated across variables that are connected by edges. This way, a causal graph reconstructs the natural mechanisms that generated the data [39]. If all edges between the variables have a clear direction and no cyclical patterns exist in the model, the graph is a *Directed Acyclic Graph* (DAG).

### Probabilistic Properties of Causal Graphs

A causal graph entails an underlying probabilistic model [40] and is therefore also called a *causal Bayesian network*. The link between probabilistic properties and causal relations is based on the *Reichenbach’s Common Cause Principle* stating that when two variables are correlated, there exists a variable that causes both variables [41]. If that causing variable coincides with one of the correlating variables, there exists a direct causal relationship between them. While a purely probabilistic Bayesian network shows merely probabilistic associations, a causal Bayesian network claims that all depicted relations are direct causal relations [42].

Bayesian networks visualize conditional independencies and conditional dependencies between variables [40]. Hereby, conditional independence means that two variables *A* and *B* are probabilistically independent only when controlling for a third variable *C*, which is denoted as *A* ⫫ *B* | *C*. On the other hand, conditional dependence implies that two variables are principally independent but become dependent when controlling for a third variable.

Each node in the Bayesian Network can be represented by a probability function feeding in the values of the antecedent nodes and returning the probability for its own values [43]. By combining the probability functions of each node, the graph can be characterized by a joint probability function. Hereby, the conditional dependencies of each node are chained together to result in a factorization formula [44]. For example, in a network *A* ← *B* → *C* → *D*, the variables *A* and *C* are dependent on *B*, with *D* depending on *C*. Using the factorization, the joint probability function can be written as *P(A, B, C, D) = P(B) * P(A* | *B) * P(C* | *B) * P(D* | *C)*. This factorization formula can be used to infer probabilities of nodes given the values of other nodes.

### Conditional Dependencies in Junction Patterns

The dependencies between variables in a causal graph generate junction patterns [45]. Figure 1 shows four important junction patterns that have distinctive implications on the probabilistic and causal relations between the variables. The simplest case is a directed edge from *A* to *B* depicting a direct causal effect. If another variable *C* lies on this directed path, the resulting pattern *A* → *C* → *B* is called a *chain*. The variable *C* that lies on the chain from *A* to *B* is called a *mediator*, propagating the indirect effect from *A* to *B*. The pattern when there exists a variable *C* that causes the other variables *A* ← *C* → *B* is called *confounding*. In this case, *A* and *B* neither have a direct nor an indirect causal link. Still, when not considering the factor *C* that causes both variables, they appear probabilistically dependent [46]. However, since controlling for *C* eliminates the dependence, *A* and *B* are said to be conditionally independent given *C*. Finally, two variables *A* and *B* could both causally influence a third variable *C*. In this case, there again exists no causal relation between *A* and *B* but since both are related to *C*, controlling for the so-called *collider* *C* introduces a conditional dependence.


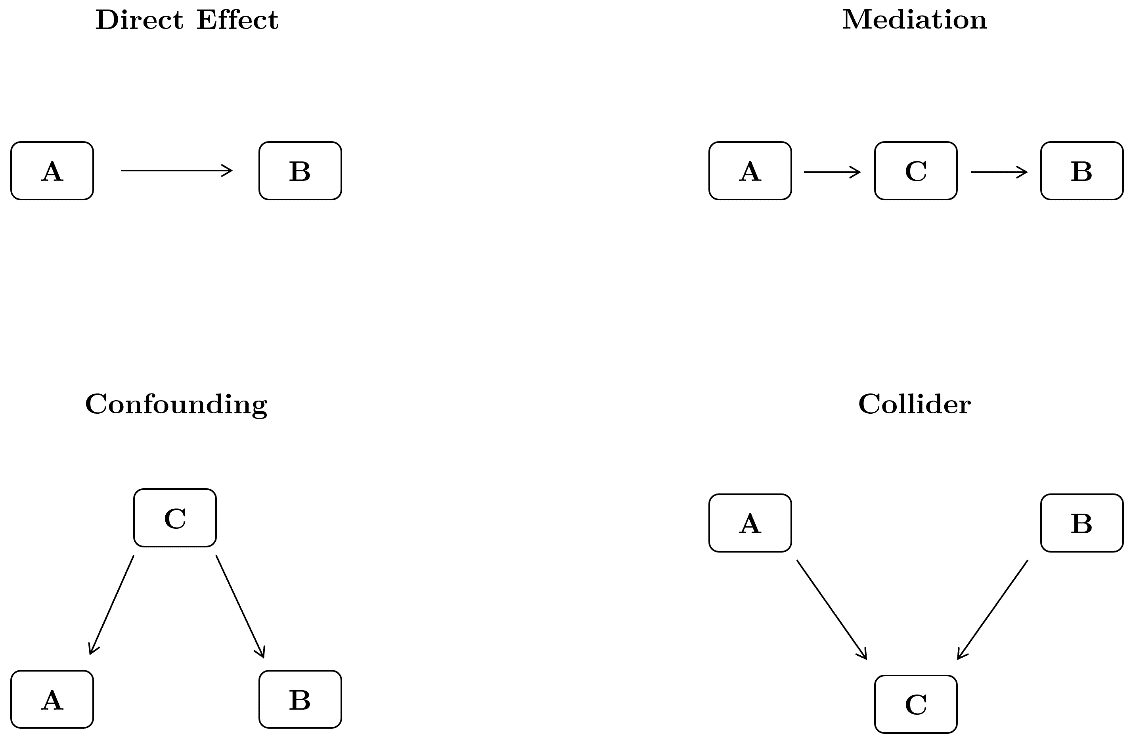


Figure 1: Junction patterns in Bayesian networks.

Conditioning on the direct parents – the nodes immediately anteceding a variable – in a chain eliminates the dependence on all other non-descendants. This statement is formalized in the *Markov property* that states that the future is independent of the past, given the present, and can be written as *X_t+1_* ⫫ *{X_t-1_, X_t-2_, ...}* | *X_t_* [43]. This property can be used to simplify the factorization formula to *P(G) = Π p(x_i_ | x_pa(i)_)* with *G* denoting the graph and *pa(i)* denoting the parents of node x_i_. Thus, by including information from specific variables, dependencies can be selectively eliminated. When this property is used to eliminate an effect transmitted on a path, this path is deactivated or *blocked*.

The notion of blocking paths can be used to read off dependencies from the graph [47]. As discussed above, a causal effect is transmitted on a chain. A path that connects variables by a chain is active and becomes inactive when conditioning on a mediator, as stated in the Markov property. Similarly, two variables that are caused by a confounding factor are dependent when not conditioning on the confounder. Hence, also the path

*A ← C → B* is active despite not containing a direct causal effect from *A* to *B*. This path is blocked by conditioning on the parent *C*. On the other hand, when there exists a collider pattern *A* → *C* ← *B*, the two parent nodes are not dependent but become dependent when conditioning on the collider. Hence, the path is principally blocked but becomes active when conditioning on *C*. To sum up, paths are active if there only exist mediators and confounders on the path and are blocked if there exists a collider [43].

To block a path, at least one mediator or confounder on an active path must be conditioned on. To unblock a path, a collider needs to be conditioned on. If all paths between two variables *A* and *B* are blocked by conditioning on *C*, this is denoted as *A* being *d-separated* from *B* by *C* and written as *A* ⊥ *B* | *C* [47].

### Observational versus Interventional Distributions

Based on the causal graph, observational and interventional distributions can be examined [48]. The analysis hitherto has been primarily concerned with observational distributions by examining probabilistic implications of conditioning on variables of an observed data set. Conditioning on observed variables corresponds to the question of how the likelihood to observe something changes, given that we observe a value of some other variable. However, since a causal graph entails causal effects that are propagated along chain structures, the effects of arbitrary interventions can also be examined [49]. To clarify the distinction between observation and intervention, Pearl introduced the *do-operator* that evaluates the value of *Y* given that a variable *X* is manually set to a value *x*, written as *P(Y* | *do(X=x))* or short *P(Y* | *do(x))* [45].

In general, the interventional distribution differs from the observational distribution [50]. This can be illustrated using the example regarding Nobel prizes from above. Let the variable *prizes* denote the number of Nobel prizes of a country and the variable *chocolate* the chocolate consumption per capita. As discussed above, the two variables are likely caused by a confounder *wealth*. Hence, *chocolate* and *prizes* are conditionally dependent given *wealth*. This dependence implies a positive correlation when inspecting the observational distribution as there exists a confounder on the path *chocolate ← wealth → prizes* that is not conditioned on and thus is active.

In an interventional setting, the conditional dependence on a confounder is eliminated because the intervened variable is exogenously set. In the present example, intervening on *chocolate* may mean that the inhabitants of a country are forced to consume more chocolate. This exogenous intervention cuts off the link between the confounder *wealth* and *chocolate*. By cutting off the connection between the confounder and the manipulated variable, the path from *chocolate* to *prizes* is eliminated. Therefore, no causal effect on the number of *prizes* results from the intervention on *chocolate*. Thus, *P(prizes* | *chocolate) ≠* *P(prizes* | *do(chocolate)).* The notion of cutting off the intervened variable from their parents is analogous to the previously discussed randomized control trials where the randomization of participants eliminates selection bias [51]. However, using causal graphs, the effect of interventions can be evaluated by only simulating the change in the graphical structure without actually performing the intervention.

The graphical implications of simulating interventions are reflected in the truncated factorization formula of the joint probability function, which shows the interventional distribution. Hereby, all terms reflecting dependencies of the manipulated variable are omitted from the factorization formula because after the intervention, the variable is no longer dependent on any other variable. This can be denoted as *P(x_v_* | *do(x_W_ = x'_W)) =*

Π *P(x_i_* | *x_pa(i))* 1 {*x_W_ = x'_W_*} *for W* ⊂ *V* [47]. Besides the removal of these dependencies, all other relationships and graphical patterns stay the same. Consequently, the truncated factorization formula can be used to calculate the pre- and the post-treatment distributions and, therefore, the total causal effect of any arbitrary intervention without actually manipulating any variable.

Analogously, the notion of blocking paths is applicable in conjunction with classical statistical tools for investigating indirect causal effects of interventions without calculating any conditional dependence term. Hereby, the paths carrying the causal effect must be isolated by blocking all other paths. In conclusion, causal graphs provide the necessary information to simulate interventions and investigate indirect causal effects from observational data by altering the graph and propagating the effect of arbitrary manipulations along causal paths.

## Workflow for the causal analysis in the study

The purpose of our study is to discover causal relationships between emotions and driving behaviour. Since there exists no standardised workflow from the raw data to the causal estimates, we developed a novel workflow that combines existing theoretical knowledge about causality. The workflow builds on the guidance for creating Bayesian models by Coyle and is based upon a causal graph [52]. The workflow is illustrated in Figure 2. As shown in the diagram, the workflow comprised three consecutive steps that were all based on a pre-processed observational data set. First, the structure of the graphical model was learnt from data. Second, the resulting mechanisms were used in the causal inference step. Third, qualitative and quantitative robustness checks were used. The insights from the robustness checks were used to improve the iterative learning process of the causal model. In the following section, each step in the workflow is presented in detail.


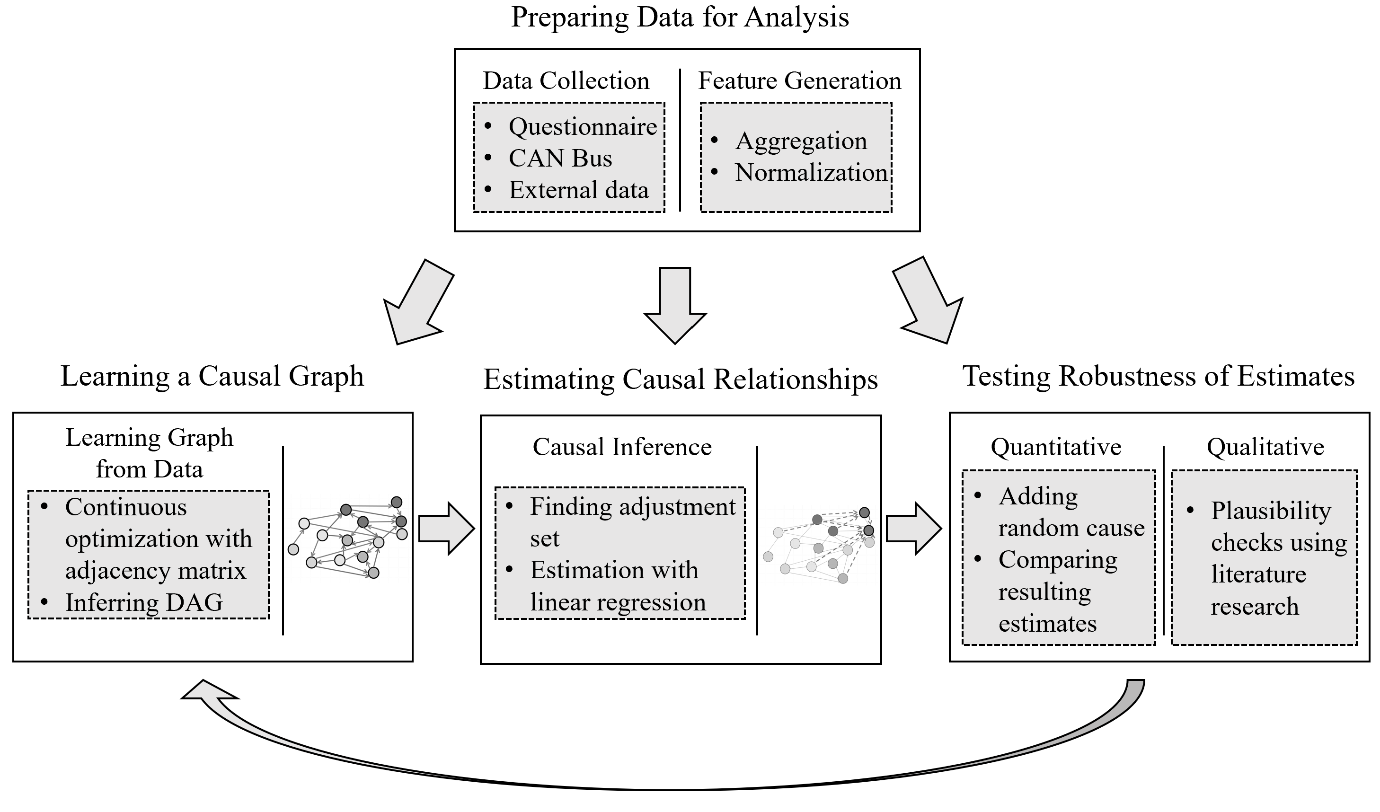


Figure 2: Our workflow for causal analysis. DAG: directed acyclic graph. CAN: controller area network (car sensor data).

### Learning the Causal Graph

In general, there are three approaches for learning a graph. First, the graph can be fully constructed from expert knowledge. Hereby, prior knowledge about mechanisms between variables is applied by including and directing edges between nodes or assigning probabilities for the existence of an effect [53]. Second, data can be used to create a graph that best fits the observed distribution and third, the prior expert knowledge can be combined with observed data in a hybrid structure learning approach [54]. Regarding well-being and driving behaviour, no complete known model of mechanisms between factors exists. Despite this lack of expert knowledge, some relationships can be *a priori* excluded, such as an effect from well-being on external factors like the weather. Thus, the causal analysis in this study was based on a hybrid approach starting with the data-based learning of a graph. Subsequently, prior knowledge was used to check the proposed edges for plausibility.

Within the category of data-based learning algorithms, it can be distinguished between constraint-based, score-based, and hybrid approaches [55]. Constraint-based methods rely on conditional independence tests for learning relationships between the variables [43]. Thus, edges are added to the graph whenever the target node is conditionally dependent on the value of the source variable. Hereby, a completed partially directed acyclic graph depicting the Markov equivalence class, which is the set of all graphs that fulfil the independence criteria for the given data, is returned [56]. Due to the reliance on conditional independence, a central assumption for constraint-based algorithms is the faithfulness condition. This condition requires all conditional dependencies to be accurately represented by the causal graph [43].

Score-based methods are concerned with finding a sparse graph that best fits the data. This fit is quantified by a scoring function. Most scoring functions are calculating the log-likelihood of the observed data given a specific graphical structure. To obtain a sparse graph, a penalization term is introduced with the *Akaike Information Criterion* using the penalization *ϕ(t) = 1* and the *Bayesian Information Criterion* using the penalization term *ϕ(t) =*
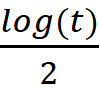
 [57]. Once a scoring method is chosen, graphical structures are evaluated, and the directed acyclic graph that had the best score is returned. With the number of possible graphs increasing super-exponentially in the number of nodes, finding the optimal structure is a challenging combinatorial problem [58].

To find the combination of edges that best fits the data, local and global search algorithms can be applied. Hereby, local search algorithms such as *Tabu-search* [59] and *Hill-climbing* [60] start with an initial guess and iteratively evolve the graph until no further improvements can be made. The *Markov Chain Monte Carlo* method builds on this idea by randomly adding or removing edges [61]. Each of those methods aims at maximizing the score function subject to the constraint that the structure fulfils the requirements for a directed acyclic graph.

A global search over all possible graphs is computationally not feasible. To circumvent the combinatorial problem, the global *DAG with NOTEARS* algorithm uses the representation of a graph *G* in form of an asymmetric adjacency matrix *W* that enables a continuous optimisation function instead of the discrete sets of nodes and edges [62]. Hereby, the combinatorial constraint *G* ∈ *DAG* is converted into a smooth inequality constraint *h(W) ≤ 0* that codes the *DAG-ness* of the adjacency matrix. Using the continuous inequality constraint, simple numerical methods can be applied to globally optimise the score. Due to this global optimisation, the algorithm was found to outperform score-based algorithms that rely on local optimisation. In comparison to constraint-based algorithms, the benefit of a score-based algorithm is that a single best-fitting graph is identified and not a set of graphs [62]. Therefore, for learning the causal graph from data in this analysis, the score-based *DAG with NOTEARS*-method was applied.

### Estimating the Total Causal Effect

The causal graph is used to estimate the total causal effect of the source variable on the target variable of interest. Hereby, the total causal effect corresponds to the change in the target variable when altering the source variable by one unit. To examine this change, all paths from the source to the target variable must be investigated. Hereby, junction patterns are of special interest as they determine which paths are active and which are blocked. Associative information is propagated along all active paths, but the true causal effect is only propagated along chains. Thus, all causally irrelevant active paths must be blocked. To block an active path, a confounder or a mediator must be controlled for, or a collider must remain uncontrolled. Therefore, the set of control variables must be identified that isolates the causal effect. This set of variables is called the *adjustment set*. Since by its inclusion, the criterion *P(y | do(x=v))= ∫_Z_ P(y* | *x,Z) p(Z) dZ* is fulfilled, the interventional distribution can be reconstructed from the observational distribution.

A method to determine the adjustment set is the *backdoor criterion*. Hereby, paths that are not solely composed of chains – so-called *backdoor paths* – must be blocked, while the chains carrying the causal effect must remain unblocked. An adjustment set *Z* is sufficient if *Z* blocks all backdoor paths from source node *x* to target node *y* and if *Z* does not contain any descendants of *x* [47]. An alternative method for isolating the causal effect is the *frontdoor criterion*. To fulfil this criterion, a set *Z* must meet three requirements. First, *Z* must intercept all chains between *X* and *Y*. Second, all backdoor paths between the source variable and all variables in the adjustment set need to be blocked, and third, all backdoor paths between the variables in the adjustment set and the target variable must be blocked by the source variable.

The identified adjustment set can be used in conjunction with classical statistical methods to estimate the total causal effect. If the relationship between the variables of interest is assumed to be linear, the coefficient *γ* of the linear regression

*E(y* | *x, Z)=α+γx+β^T^ Z* from the source variable *x* to the target variable *y* while controlling for the adjustment set *Z* yields the total causal effect from *x* to *y*. Besides linear regression, other statistical methods, including machine learning algorithms, can be applied to estimate the total causal effect. The workflow used the backdoor criterion to determine the adjustment set whenever possible. In the case when the backdoor criterion was not sufficient, the frontdoor criterion was applied. To maximize interpretability, a linear regression controlling for the adjustment set was used for estimating the total causal effect.

### Testing the Robustness of the Estimates

To evaluate the robustness of the estimate for the total causal effect, quantitative and qualitative tests can be applied. Regarding quantitative tests, the sensitivity to changes in the causal graph can be measured. One way to do so is by adding additional random variables to the graph, which should not affect the estimates. Alternatively, the values of existing variables can be changed. For instance, when replacing the values of the source or the target variable with random values, the causal effect should go to zero. Finally, the sensitivity of the estimate can be measured by randomly subsetting or bootstrapping the data set. Hereby, the resulting estimates should be close to the estimate from the total data set. Regarding qualitative robustness tests, the plausibility of the resulting direct and indirect effects can be evaluated using prior knowledge. Implausible relationships can be listed for exclusion in the next iteration of learning the causal graph. In the proposed workflow, both quantitative and qualitative robustness tests were applied.

## Implementation of the workflow

Before starting the iterative process for causal discovery, the data had to be pre-processed. Since a potential limitation of the *DAG with NOTEARS* algorithm for structure learning is the sensitivity to scale and variance [63], all variables were standardized to a mean of 0 and a standard deviation of 1. The variables of the category *emotions* were additionally standardized by each driver because the mean and the variance differed significantly between drivers. This variation is likely attributable to the personal style of assessing well-being rather than to the driving experience. Thus, standardising per driver eliminated this subjective component.

Learning the causal graph was the first step in the causal workflow. As mentioned in the previous section, the *DAG with NOTEARS* algorithm was used for this task. The algorithm was implemented in Python using the package *CausalNex* [64]. Edges below the threshold of 0.1 were removed. With this algorithm, a directed acyclic graph was constructed. This resulting structure was converted from a proprietary format to the open *GML* format using a self-coded function.

The inference step was implemented in the Python package *DoWhy* [65]. The causal estimate was calculated for all pairs of variables that lay on a chain connecting to variables of the category *emotions*. First, the adjustment set was determined using the backdoor criterion. This criterion was sufficient to determine a valid adjustment set in all examined causal relationships. Second, the causal estimate was calculated using a linear regression controlling for the identified adjustment set. The last step of the proposed workflow was the robustness test of the model. For the quantitative evaluation, a random confounder was added to the graphical structure using the package *DoWhy*. By comparing the resulting estimate to the original one, the sensitivity to changes in the graph was assessed. The qualitative plausibility check was conducted on the direct causal effects in the Bayesian model. Based on prior knowledge, implausible relationships were added to the list of *a priori* blocked direct causal effects, which is shown in Multimedia Appendix 5. After a relation was added to this list, the entire workflow was repeated.
