# Supplementary material for: Understanding the Interactions Between Driving Behavior and Well-being in Daily Driving: Causal Analysis of a Field Study
Source: J Med Internet Res. 2022 Aug 30;24(8):e36314. doi: 10.2196/36314 (PMC9472037; doi:10.2196/36314)
Supplement: Multimedia Appendix 5 [file jmir_v24i8e36314_app5.doc]

## Multimedia Appendix 5: A priori excluded Direct Effects

The table below shows the direct causal effects that we excluded a priori in the creation of the causal directed acyclic graph due to impossible chronological order or being an implausible effect. For each effect, we indicate the reason for its exclusion.

| **Source** | **Target** | **Reason for Exclusion** |
| --- | --- | --- |
| After Arousal | Before Arousal | Implausible chronological order |
| After Arousal | Speed | —"— |
| After Arousal | Flow | —"— |
| After Arousal | Steering | —"— |
| After Arousal | Sun | —"— |
| After Arousal | Sudden Events | —"— |
| After Arousal | Braking | —"— |
| After Arousal | Before Valence | —"— |
| After Valence | Before Valence | —"— |
| After Valence | Before Arousal | —"— |
| Steering | Before Arousal | —"— |
| Steering | Before Valence | —"— |
| Speed | Before Arousal | —"— |
| Before Arousal | Sun | Implausible Effect |
| Before Arousal | Flow | —"— |
| Braking | Flow | —"— |
| Speed | Flow | —"— |
| Commute | Sun | —"— |
| Occupants | Weekend | —"— |
| Steering | Length | —"— |
| Braking | Length | —"— |
