# Supplementary material for: Understanding the Interactions Between Driving Behavior and Well-being in Daily Driving: Causal Analysis of a Field Study
Source: J Med Internet Res. 2022 Aug 30;24(8):e36314. doi: 10.2196/36314 (PMC9472037; doi:10.2196/36314)
Supplement: Multimedia Appendix 6 [file jmir_v24i8e36314_app6.doc]

**Multimedia Appendix 6: Data-based causal DAG**

The figure below shows the causal directed acyclic graph (DAG) that was learnt from the data from the field study using the DAG with NOTEARS algorithm. The nodes represent the variables included in the model, the edges indicate direct causal effects between variables.

**
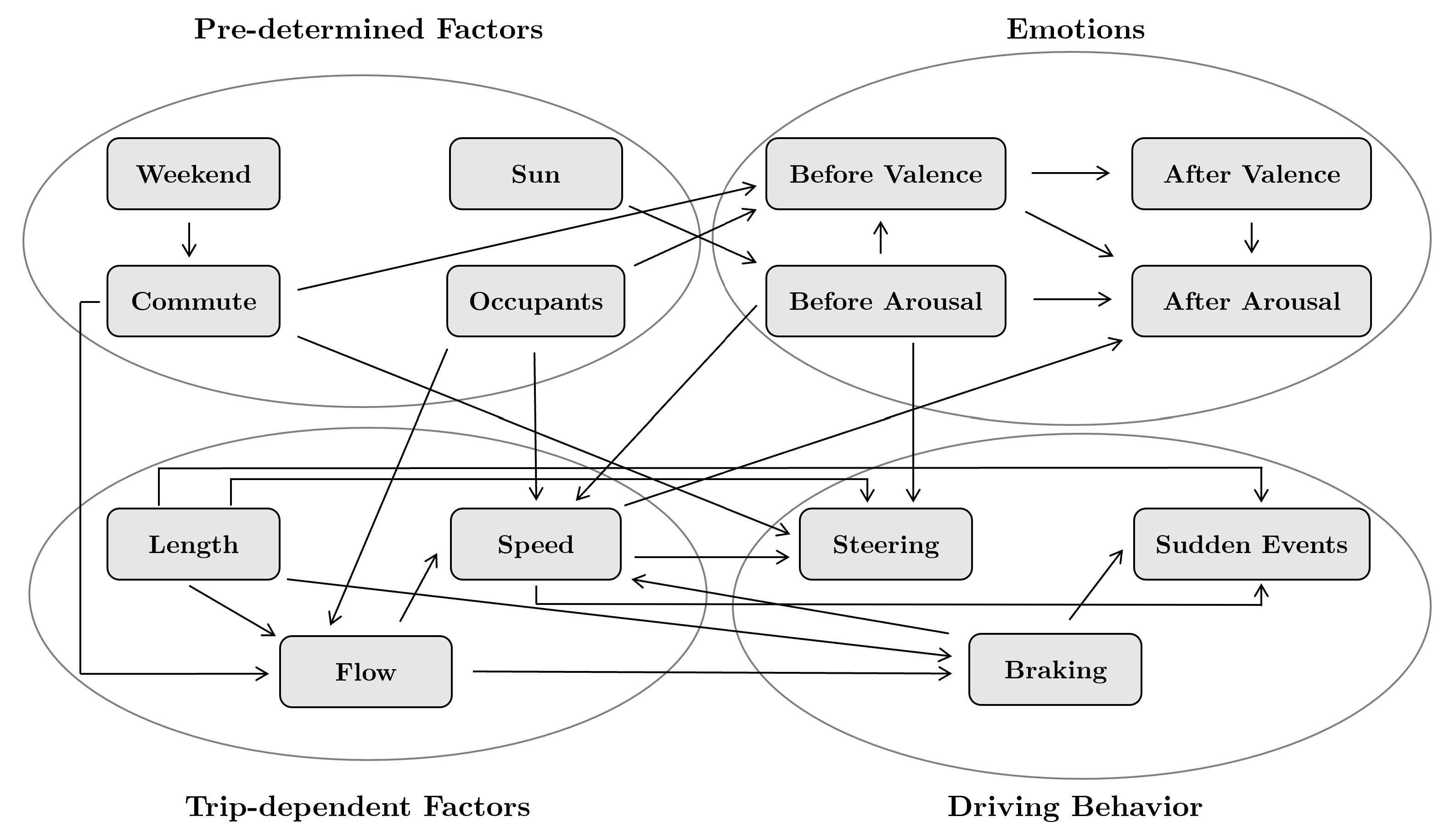
**
